# Mapping the Swiss Vaccine Supply Chain

**DOI:** 10.3389/fpubh.2022.935400

**Published:** 2022-07-18

**Authors:** Bublu Thakur-Weigold, Pascal Buerki, Patrice Frei, Stephan M. Wagner

**Affiliations:** Department of Management, Technology, and Economics, ETH Zurich, Zurich, Switzerland

**Keywords:** COVID-19, vaccine supply chain, supply chain mapping, GIS, Switzerland

## Abstract

**Objectives:**

The design of the supply chain determines how effectively any vaccination campaign can be operated. This case study of Switzerland's vaccine supply chain compares its design with public health objectives. It maps the vaccine supply chain in Switzerland as it was set up to handle the first shipments of Covid-19 vaccine in 2021 to enable a more holistic view of supply and demand flows. Recommendations are made to improve emergency logistics of vaccines in the future.

**Methods:**

Twenty-six semi-structured interviews with international and Swiss stake-holders were coded and analyzed to arrive at a description of planning and distribution processes. The vaccine supply chain network structure was mapped, linking upstream and downstream flows of material and information. The visualization of nodes and flows was combined with spatial information, including population data. The results are summarized in narrative form to support decision-makers across disciplines.

**Results:**

Despite adequate vaccine supply, abundant local endowments and high investment in infrastructure, the 2021 design of Switzerland's vaccine supply chain reduced the potential reach of target populations. The segmentation of catchment populations, collaboration between administrative units and better use of information on geolocation and material flows could have improved the speed and reach of vaccinations during the emergency response phase. Three recommendations are made for supply chain structures to support higher vaccination rates in the future.

**Conclusions:**

The visualization identifies design alternatives which could have improved vaccination rates under the prevailing conditions. A supply chain map provides public health officials with a shared view of the vaccine supply chain in order to better match supply with demand. The case study contributes to developed country studies. In order to improve public health outcomes in Switzerland, investments to secure supply, strong national endowments, and excellent infrastructure must be combined with optimized supply chain design.

## Introduction

**Figure 1:**
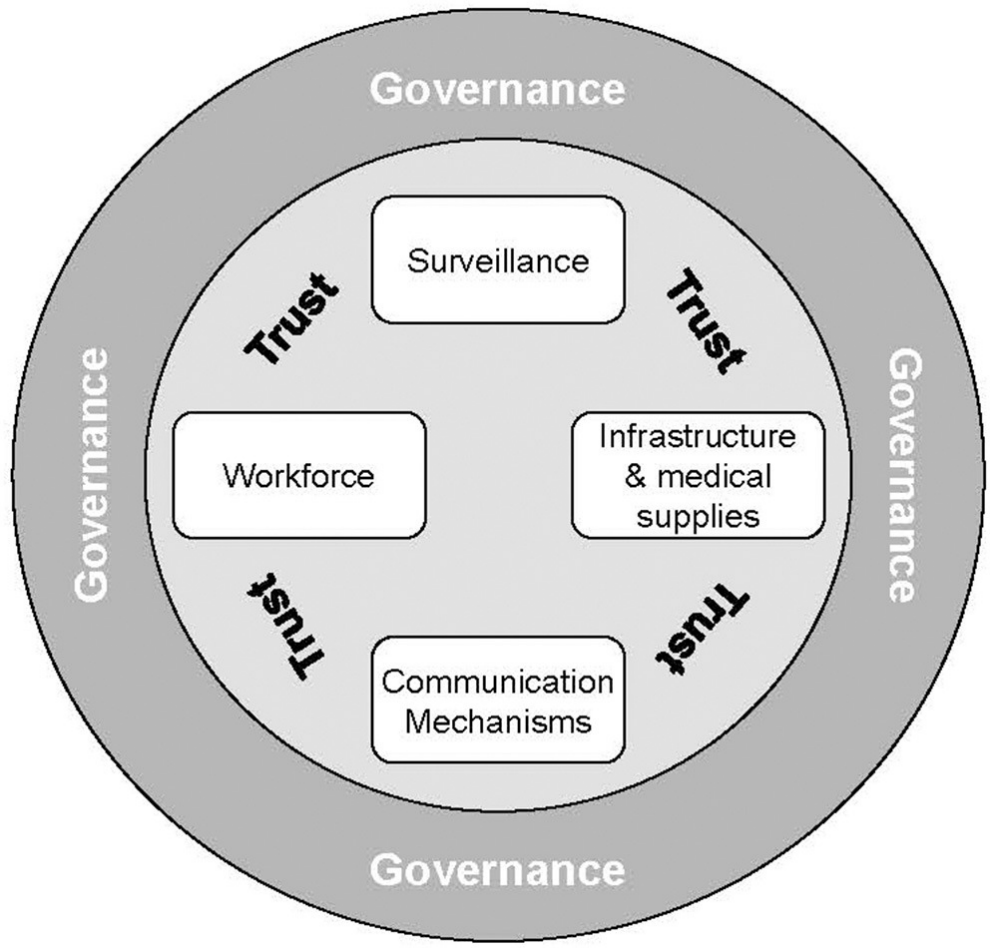
Model of the elements of preparedness for emerging infectious diseases derived from a synthesis of the public health literature. Source: (5).

In the fight against Covid-19, much attention was paid to the development of the vaccine itself, an extraordinary breakthrough in the fight against the virus, but arguably only the first step in national campaigns against the pandemic. A variety of global experts have published guidelines for emergency vaccine logistics, but these vary widely in their scope and approach. According to a case study of “the global” Vaccine Supply Chain by the INSEAD-Wharton Alliance, enormous public investments have been made into the development of an effective and safe vaccine, as well as agile manufacturing capacity to serve the world population (1). The need for infrastructure, skills in batch management and access to intellectual property are acknowledged (2). The WHO convened a task force whose mission was to “establish a COVID-19 supply chain system (CSCS) to provide countries with essential supplies needed for their COVID-19 response” [(3), p. 1] effectively focused upon the procurement and allocation processes, leaving “regionalization” and in-country distribution to local health authorities. The stated intent of WHO's global vaccine supply chain was to “deliver essential supplies to where they are needed the most”. An after-action assessment questioned, however, the extent to which this strategic objective of equitable distribution was achieved. One of the shortcomings it identified was the lack of an end-to-end supply chain strategy [(4), p. 15]. Palagyi et al.'s review of studies of low-income countries (5) proposes a conceptual framework for Emergency Infectious Diseases (EID) preparedness, comprised of six health system constructs, four of which are “hardware” (Surveillance, Workforce, Infrastructure and Medical Supplies, and Communication Mechanisms), and the remaining two “software” (Governance and Trust), see Figure 1.

Public health experts have identified key aspects of emergency preparedness. However, within their checklists, logistics design remains generic, assumed to be some combination of manufacturing, storage, and transport infrastructure, plus material availability. The existing tools and framework are a step toward a more systemic approach to *what* must be prepared, but do not provide guidance on *how to* do so optimally. It is here that the supply chain literature can fill gaps.

We begin by suggesting that public health authorities manage the entire “hardware” system, beginning with the sourcing of raw materials which flow into the manufacturing plants, where they are converted into the finished product and readied for distribution. Since it is not vaccines which save lives, but vaccinations (6), the finished doses must be moved locally to reach the arm of the patient. For this, every country needs to set up a supply chain (7), which is “*the ecosystem of organizations, people, technology, activities, information and resources that have to come together to ensure the delivery of the product from the point where it is manufactured to the end-patient in a cost-effective way*” [(8), p. 142]. There are other strategic objectives which must be met. Since no one is safe until everyone is safe (9, 10), in an emergency the system must maximize the number of vaccinations in the shortest period of time. Only then does the vaccine supply chain become an enabler of herd immunity in any country.

To add to the extant research on developing countries (5), the objective of this study is to diffuse learnings from the case of a rich country setting up the hard- and software of its vaccine campaign. Switzerland is arguably the world's richest country, with the highest GDP per capita with a population smaller than New York City. Economic data confirm that the rebound of domestic economic activity in 2021 was partially fueled by the growth in pharmaceuticals, both during and after the pandemic lockdown. Since its firms participated strongly in the production and global distribution of the Covid-19 vaccines (11), this country should possess the prerequisites for a high-performing supply chain: pharmaceutical research capacity, domestic manufacturing capability, and infrastructure for distribution.

It was not, however, initially evident that Switzerland's endowments like best-in-class healthcare, infrastructure, and public transport systems would ensure that the last citizen in the most remote valley would be vaccinated promptly. The mapping project was launched in the midst of the early waves of infection in February 2021, at a time when there was no clarity of how the rollout of a new Covid-19 vaccine would proceed in Switzerland. It was also unclear whether the slow ramp up of vaccinations was attributable to supply challenges, bureaucratic issues, or other root causes. Health offices, pharmaceutical companies, medical professionals (in both private and public practice), hospital administrators, politicians, journalists and, not least, the public were all functioning with a partial and incomplete (siloed) view of the vaccine production and distribution system. Influenced by public discourse and an increasingly vocal press, individual components (like vaccine development or manufacturing), of the supply chain were being discussed in isolation, usually as inputs for political, rather than operational decisions. This investigative study resolved to connect the dots and construct the overview of the end-to-end supply chain which, at the time, no single entity, stakeholder, or decision-making body possessed. Its motivation is to support especially the medical professionals and public administrators who are decision-makers in national supply chains, but are not routinely trained in supply chain management.

## Context and Methods

### Switzerland's Healthcare System and Pandemic Preparedness

Switzerland has a high-performing healthcare system (measured by indicators like life expectancy), with mandatory coverage for all residents. It is also one of the most expensive, with a health expenditure per capita in 2013 of around 6,187 USD, compared to the EU average of 3,379 USD (12). Research (12–14) characterizes the Swiss healthcare system as complex and highly decentralized, organized according to the traditional administrative structures in which the federal government plays a regulatory role, defining the system of financing, the product safety and quality assurance, and policies for public health. It is the 26 cantonal governments which are responsible for providing the healthcare services for their individual populations. Their responsibilities include the planning and operation of hospitals, administration of nursing homes, emergency services, control of pharmaceuticals, and disease prevention. Independent operations in each canton have produced a heterogeneity of healthcare services nationwide (13, 14). Since cantonal planning also determines management practices and purchasing efficiency, De Pietro et al. (12) express concern about the oversupply and redundancies in hospital services. Health reforms in the past decades show a trend to increased intervention at the federal government level to coordinate tasks, the equivalent of a more centralized approach.

To ensure that everyone has access to standard healthcare services (like primary care), each resident of Switzerland is obliged to take out a health insurance policy, and insurers are not permitted to reject applicants. However, the premium may vary, depending on the place of residence and, to a certain degree, the age of the insured person. Cantons support their poorer residents with subsidies, although the amount of the subsidy, and the conditions under which these are available, again differ from canton to canton, with each administration defining its own rules. As a consequence, two otherwise comparable patients of the same age and income, but living in two different cantons, may not necessarily get the same support (12).

Beside differences in financial support, because there is no central regulator who steers the development of the healthcare system in Switzerland, the distribution of services varies substantially, both between and within cantons. For example, in 2000, the canton Basel-Stadt had four times more primary care per capita than the canton of Appenzell Innerrhoden. These variations arise partly from agreements between cantons to collaborate (12). In spite of the regional differences in service density, the general accessibility to care in Switzerland is considered to be very good, attributable to the high quality of the country's public transportation system.

In 2018, Switzerland had developed a preparedness plan for an influenza pandemic which defines the roles, responsibilities and interactions of the relevant stakeholders and provides a guideline for the cantons for their own pandemic plans (15, 16), see Table 1.

**Table 1:** Stakeholders and responsibilities in a pandemic, based on BAG (15, 16).

**Stakeholder**	**Responsibilities**
Federal Office of Public Health (FOPH) (Bundesamt für Gesundheit, BAG)	Development of strategy for procurement and national supply. Determination of risk groups and prioritization of vaccine allocation (recommendation only). Definition of a distribution key and calculation of quotas to the cantons. Steering and coordination of supply.
Federal Commission for Questions of Vaccinations (Eidgenössische Kommission für Impffragen, EKIF)	Development of a vaccination plan and collaboration with the FOPH for strategy development and the choice of vaccines.
Swissmedic	Approval of vaccines.
Pharmacy of the Swiss Armed Forces (Armeeapotheke, AApot)	Procurement, logistics and, if required, storage of vaccines. Monitoring and ensuring distribution/delivery of vaccines along the entire logistics chain (logistics monitoring). If required, re-packaging and destruction of excess supply.
Cantonal government	Logistics within their territory. Control and assurance of distribution within the canton in line with demand of the canton. Implementation of the vaccination campaign. Destruction of surplus vaccines stored in the canton. Enactment of compulsory vaccination for vulnerable groups of the population.

In 2018, the Federal Office of Public Health (FOPH or BAG) also mandated a study to determine how best to allocate scarce supplies of vaccine in a fair and equitable manner, as well as identify which populations should be prioritized to ensure an effective intervention. The study (17) modeled different scenarios and allocation principles using the population data of Switzerland. It recommended a pro-rata approach based on the size of the prioritized subpopulation, as well as the setup of a database to visualize the demand and supply of vaccine stockpiles, combined with details of the canton, age and risk groups, professions, and more. Such a database would allow an optimal management of the vaccine campaign by providing decision makers essential information about the vaccination coverage (e.g., number of people vaccinated in the prioritized group), in real time, enabling a swift and flexible response.

Based on the national pandemic preparedness plan (16), the cantonal governments proceeded to establish their own plans which defined cantonal pharmacists as responsible for logistics, as well as the key distribution channels, ranging from mass vaccination centers, to primary care physicians and mobile teams (18). These cantonal plans were, however, made on an aggregate level, which did not specify the number of distribution points or their locations. These details were to be worked out after the outbreak of any particular infectious disease (18–21), and were soon put to the test by the outbreak of Covid-19.

### Supply Chain Mapping

The research asked whether the Covid-19 vaccine supply chain was designed to maximize vaccinations quickly, and whether the necessary information flowed to support the performance of the public health interventions. Furthermore, were stakeholders working together and not leaving functional specialists to pursue local objectives to the detriment of system performance? The methodology combined strategic supply chain mapping of the network structure as described by Gardner et al. (22) which established a shared view of the system, whose performance was then evaluated using defined supply chain drivers (23, 24). The choice and details of these methods are as follows.

The strategic supply chain mapping focused on the network structure, nodes, and spatial or geographic locations (22, 25), in order to create a visualization of the system participants, as well as flows of material and information. The emphasis upon linkages in the dependent system is intended to counteract siloed and functional decision-making (22), both of which impair overall performance, but are rational and intuitive behaviors. Process or value-stream mapping was not attempted in the project scope.

**Figure 2:**
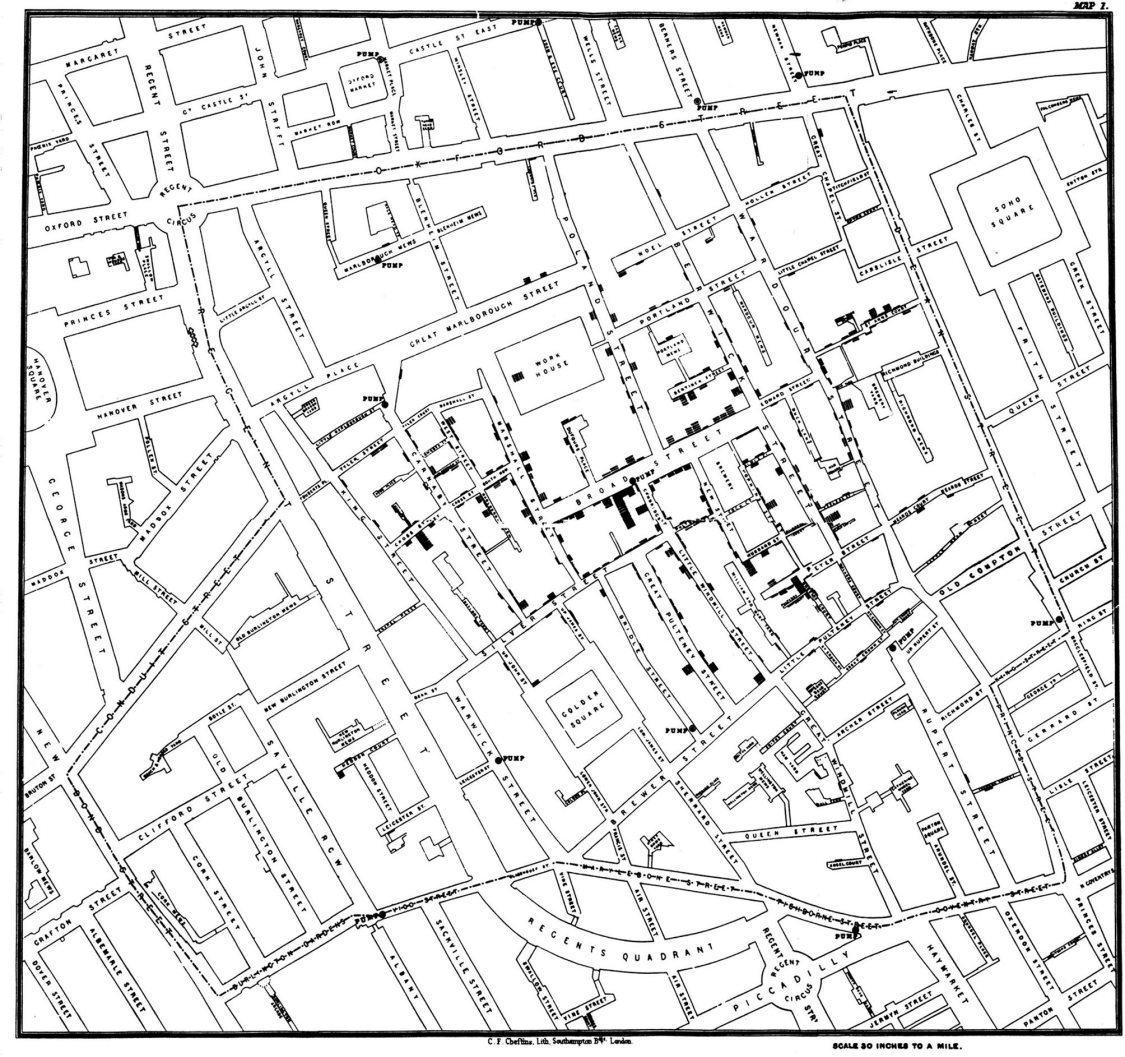
“There were many persons who drank the water from Broad Street pump about the time of the outbreak, without being attacked with cholera; but this does not diminish the evidence respecting the influence of the water…The deaths which occurred during this fatal outbreak of cholera are indicated in the accompanying map” [(26), p. 45]. Source: https://de.wikipedia.org/wiki/Datei:Snow-cholera-map-1.jpg.

The decision to include spatial data was made to support epidemiological insights. It was inspired by the study of the Soho cholera epidemic carried out by Dr. Snow in 1854, who discovered by mapping cholera infections that the caseload was distributed close to a single well at the epicenter of the outbreak (26) (Figure 2). All the patients had drunk water from this source, which later proved to be contaminated by a sewage leak. When the removal of the pump's handle halted the contagion, Snow concluded that the pathogen was not in fact transmitted by noxious gases, according to the predominant “Miasma” theory of the time, but waterborne.

Snow's recognition of the spatial relationship of the cholera case data confirms the first law of geography, which states that “Everything is related to everything else, but near things are more related than distant things” [(27), p. 236], and the key to his insight was visual mapping.

**Figure 3:**
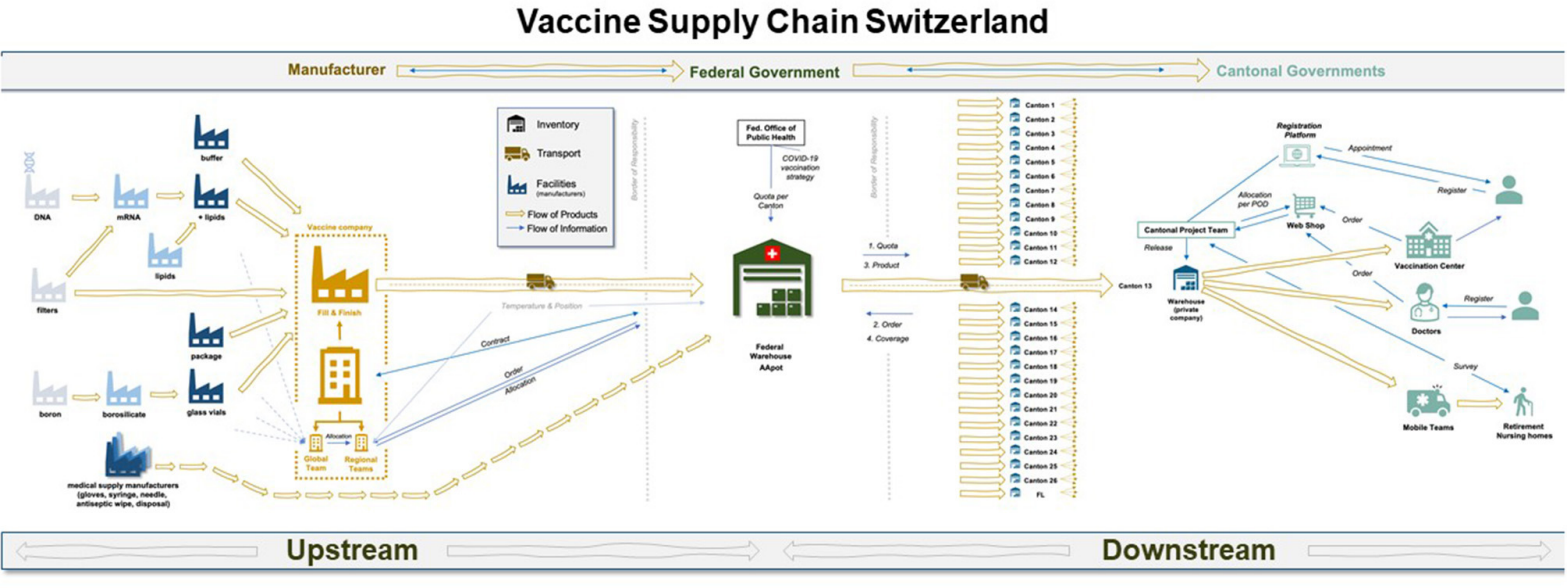
Map of the end-to-end vaccine supply chain in Switzerland, as it was configured in 2021. The upstream section roughly begins with raw material suppliers and leads to the central warehouse managed by Swiss armed forces in a classified location. The downstream segment begins at the central warehouse for vaccine stockpiles to the arm of the patient. Source: authors.

The network map was logically divided into two segments (illustrated by Figure 3), each with its own decision-makers and interest groups: *Upstream* (Supply and Production), and *Downstream* (Distribution and Demand). The upstream segment reflects the global nature of vaccine production with inbound flows of finished doses into Switzerland, while the downstream supply chain, which begins at the federal pharmaceutical warehouse and ends in the citizen's arm, is situated within the Swiss public healthcare system.

To assess the extent to which the identified network structure met its declared strategic intent, the study focused on four supply chain performance drivers from the literature.

First of all, each supply chain has a *design* which supports the strategic purpose of its organization (23). Contrary to the popular discourse which often speaks of ***A*** global supply chain [see also (1)], there is no single generic design for supply chains which ensures high performance in all business cases. In other words, one size does not fit all. Each supply chain must be designed to fit its strategic purpose and uncertainty profiles (28, 29). The Swiss vaccine supply chain will necessarily be different from its U.S., Indian, or South African counterpart. A misfit between purpose and design results in poor performance (30), which can usually not be compensated by reactive operational measures (like increasing input resources, cutting costs, or working overtime). It is therefore necessary to distinguish between supply chain *design*, which comprises both the physical and logical set-up (including process configurations, and the business rules of the system), and its *operations*, which are the execution of that design.

Second, although supply chains are commonly associated with the movement of goods, the material flows are only possible if the *flow of information* between decision-makers is smooth and unbroken. These include order quantities, inventory levels, and demand or consumption forecasts, all of which must be shared in a timely, complete, and accurate form.

Third, effective emergency response inevitably involves multiple decision-makers, who must collaborate to deal with the prevailing conditions of uncertainty (31).

Fourth and finally, although it appears intuitively correct to do the best job for any single function or node in the chain, local optima do not necessarily coincide with optimal system performance. This means that independent decision-making in a dependent system (even if this is intuitive and rational behavior for specialists), tends to reduce the overall system performance (32).

The input data for both the network mapping and its performance drivers were collected in the form of 26 semi-structured interviews with subject-matter experts from public and private sectors, as well as decision-makers in Switzerland (Table 2).

**Table 2:** Key informants interviewed during the course of the study.

**No. of**	**Functional location of**	**Informant's area of**
**informants**	**Informant**	**expertise and interest**
1	Lecturer and Researcher at INSEAD	Theory and practice of international vaccine supply chain management
2	Program Director at US Pharmaceutical firm	Vaccine program management in the pharmaceutical industry
2	Business Unit Lead Switzerland Market Lead Switzerland	Covid-19 vaccine manufacturing
7	Swiss Federal Office of Public Health, Swiss Department of Defence	National public health management, emergency response
8	Cantonal offices	Municipal and local administration
1	Swiss Vaccination Center	Patient-facing vaccine administration at cantonal level
5	Retail industry, Academic Research, Swiss Armed Forces	GIS technology

Each interview took approximately 1 hour. After asking each informant to describe their current role within their organization, the interview questionnaire was structured into two decision areas: allocation (decisions about prioritization and equity), and distribution of vaccines (the network which provided access to the vaccines). Being presented with a graphic visualization derived from the inputs of the vaccine supply chain stakeholders, the geographic information systems (GIS) specialists were asked to familiarize themselves with the structure of the Swiss Covid-19 vaccines supply chain, then requested to provide their thoughts on the possible use of GIS to address the known challenges. Each interview was recorded and transcribed. The resulting dataset underwent a structured content analysis using MAXQDA software according to Kuckartz (33).

Publicly available geospatial data and GIS were retrieved to provide the relevant situational awareness [(25), p. 3]. Together, these datasets were tapped to arrive at a description of the vaccine supply chain system in early 2021. The performance assessment has been synthesized here in narrative form (34), to facilitate cross-disciplinary collaboration without requiring specialized technical knowledge.

## Results Of Mapping

### The Upstream View

The first half of Figure 3 makes explicit the multiple flows of information and material in the upstream vaccine supply chain, ending in the federal warehouse in Switzerland. The complexity of operations is accordingly high, as 280 ingredients are directed through the international network toward a fill and finish site in Europe (Belgium for Pfizer and Spain for Moderna), after which they are prepared for transport into Switzerland.

New products have an uncertain supply base, as well as unstable manufacturing outputs, because standard processes do not usually exist at the outset. Each sub-process is evolving, being tested, and adjusted to learnings and changing boundary conditions. The Covid-19 vaccine was no different. Sourcing issues, shifting bottlenecks and capacity constraints all emerged during the system ramp-up (1). These included shortages of Borosilicate (a raw material required to make glass vials), the lipids in which the active ingredient was encased, or simple medical commodities like syringes. Not only was raw material in short supply, but the parts needed to produce the vaccine, like specialized filters, proved difficult to procure. Engineers had to decide whether the best packaging form was 5/6 or 10/11 doses, a judgement call which could have serious consequences not only for the cost per dose, but waste if too few patients presented themselves for vaccination after the package of perishable doses was opened. The vaccine supply chain would have to be designed to deal with these supply uncertainties, while building urgently-needed capacity in markets which were besieged by global demand in 2021 (28, 29).

Switzerland joined the rest of the world to initially prioritize the assurance of its domestic supply. As the supply chains were set up at the same unprecedented “warp speed” (35), at which the vaccine was developed, purchasing governments had no upstream visibility. The supply that was allocated to them was communicated only 2–7 days before actual delivery, and local facilities had no choice but to be prepared. On the ground, approval processes in collaboration with regulatory institutions, like Swissmedic, were accelerated accordingly.

### The Downstream View (in Switzerland)

Once the vaccine supply had arrived in the central federal Army warehouse, planning and distribution processes were necessary to reach the physical patient. The following section describes the design of the network of vaccine centers, and how many doses to preposition at each of these locations. These are classic supply chain design decisions which should be aligned to the strategic intent of the system. The performance of the system will depend upon how the trade-off between cost (in the form of inventory and capacity), and responsiveness is managed. In the case of emergency vaccine response, speed is of the essence and cost was apparently no object to most developed nations, which were spending freely to restore economic growth (36). The objective was to maximize vaccination rates at high speed, and our mapping exercise identified new challenges to achieving it.

As described in the Section on Switzerland's Healthcare System and Pandemic Preparedness, the Swiss healthcare system has some of the best outcomes, but is also one of the most expensive in the world (12). It is highly decentralized because the 26 cantons are granted sovereignty over their territories. The 26 cantons vary strongly in population size: the smallest has 16,000 inhabitants compared to the largest with about 1,500,000 residents. The vaccine rollout perpetuated the historical sovereignty (13, 14), as each of the Cantons built up local proprietary capacity, planned, and distributed independently of one another. Some high-level dialogue between cantons took place, but each effectively focused on its own territory without collaborating systematically with its neighbors.

**Figure 4:**
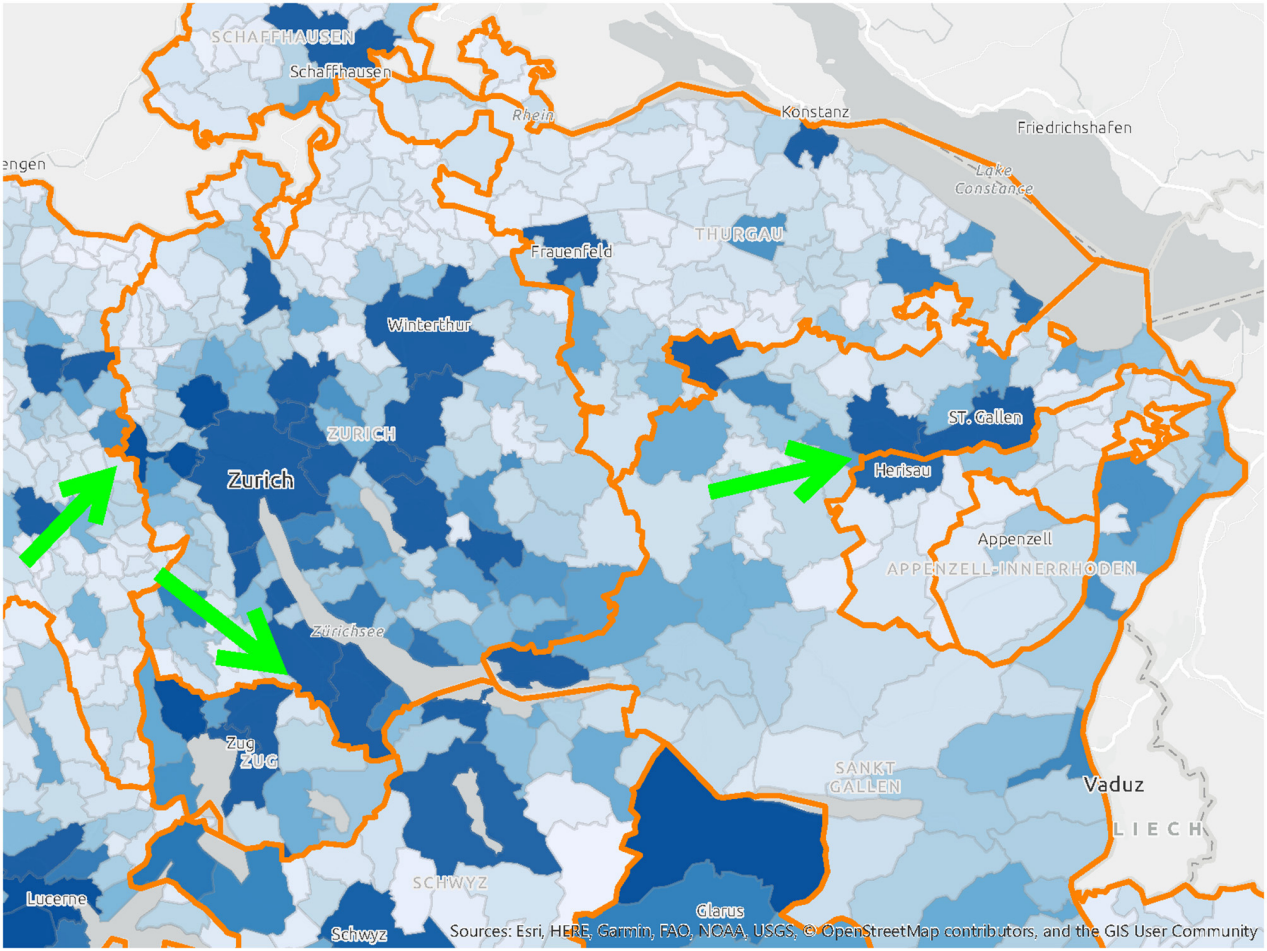
A map of population distribution and cantonal borders in Switzerland, highlighting the areas where these borders cut through high-density areas. Source: drafted by authors from data sources (37), Basemap: Esri.

**Figure 5:**
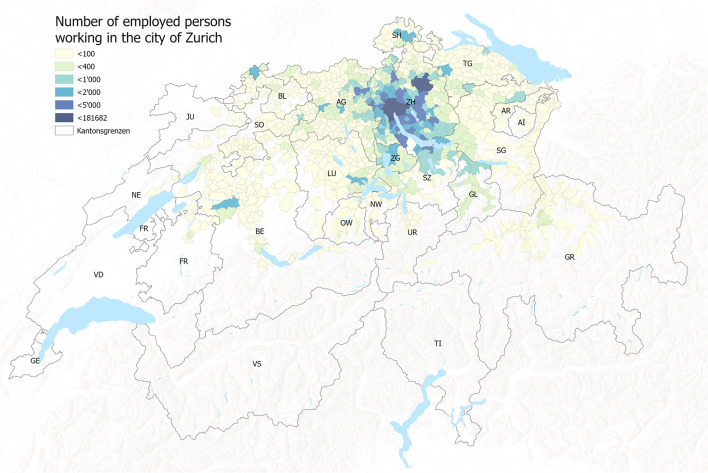
Commuter catchment area for the city of Zurich, illustrating the fact that commuters regularly come and go from more than 11 different cantons. Source: (37).

**Figure 6:**
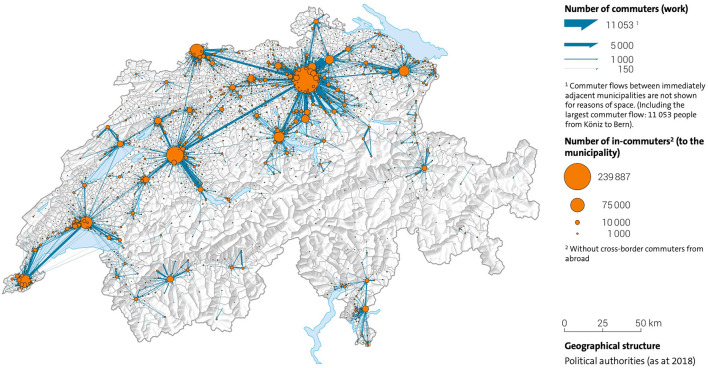
The most important commuter flows in Switzerland 2018 in relation to major urban centers. Source: (43).

It was assumed that citizens would be vaccinated close to their homes and in their canton of residence. These do not, however, actually match the residential population density (Figure 4), nor do they account for daily commuting trajectories of that population (Figure 6). The agglomeration areas of major cities like Zurich or Basel do not end at the administrative boundaries of the Swiss cantons (Figure 5). Residential distributions cross cantonal borders, and residents of one canton could find themselves in closer proximity to the vaccine offering in another canton, either because people work on the other side of, or because they live near the cantonal border. It became apparent that a portion of the population would have preferred to be vaccinated where they spent most of their waking hours, in other words at the place of their work. Dosage quotas were, however, allocated to the cantons in proportion to the size of their registered resident population, which led to potential mismatches of supply and demand.

In general, there was little evidence of deliberate planning of the last mile logistics, and distribution priorities were segmented by age and medical risk groups only. In the cantons, vaccination centers were mainly placed near existing health facilities, or based on rough-cut approximations of physical distances within the administrative territory. The participation of primary-care physicians to reach the elderly was made voluntary. At the patient level, the Swiss vaccination program was organized into medical target groups based on age and health pre-conditions. An early analysis of FOPH Covid-19 data (38) showed that people with a low socioeconomic status tend to suffer more from infections. These groups were not identified and targeted, although their place of residence and work tend to be clustered and recorded by public data. According to the interviews, non-medical characteristics of the target patient groups (e.g., socioeconomic status, religion), or those which are “hard to vaccinate” (39) were not considered. Just because someone is physically close to a vaccination center, does not guarantee they trust in the modern medicine. In other words, the selection and location of vaccination centers at the time of the study did not account for factors which influenced both willingness and opportunity to be vaccinated. Furthermore, public data could have been better used to support evidence-based decision-making in the vaccine supply chain. While it is common practice in Switzerland to use its data on private citizens for managing fiscal revenue, it is presumably not tapped on a routine basis to analyze the relevant dimensions of public health.

The operational differences within individual distribution channels of one canton (e.g., the progressive integration of primary-care physicians or pharmacists, or the opening hours of vaccination centers) but also within all cantons, resulted in varying speeds of vaccination coverage. The data suggest that the independent planning and execution of 26 local governments, which fragmented resources, capacities, planning, and response, capped the performance of the Swiss vaccine supply chain at a suboptimal level, as predicted by Yadav (8).

As the supply chain was being set up by this logic, the public was bombarded by media reports. These influenced not only the perception of vaccine efficacy and risk, but also turned the comparative availability of vaccines within individual cantons into a populist debate. The perceived differences of the speed incentivized citizens to register for vaccinations in cantons other than their own place of residence (where they were counted for supply quotas). The fact that citizens shopped around the region for the most convenient appointment with the most likely availability drove a potential mismatch of supply and demand.

## Discussion

Much attention has been paid to “*the*” vaccine supply chain in the world, especially supply and take-up, vaccine resistance, misinformation, and political actions which has resulted in highly variable capacities of individual nations to vaccinate their populations (36, 40, 41). At the time of writing, vaccine supplies in Switzerland exceed all possible demand scenarios, while actual vaccinations remain well below international benchmarks like Portugal. We therefore conclude that during the first series of vaccinations in 2021 the extraordinary investment in the Swiss vaccine supply chain did not achieve its strategic objective.

Next, we consider the root causes. There were few major issues of supply, although the understanding of inbound material flows could be improved. For example, before the vaccine arrives in the federal army pharmacy, GIS data could have made the unpredictable supply flows of a new vaccine more transparent. Real-time tracking of freight is already being used by logistics providers to ensure an unbroken vaccine cold chain, and this visibility could mitigate supply risks up through several tiers of the inbound chain. It could also enable better planning of the high-security transport capacity and routes to the secret national federal warehouse. This solution would improve the information flows and collaboration in the supply chain.

It is likely that legitimate concerns about global supply diverted administrative attention from the problem of how to manage local demand. There were extenuating circumstances, like public health regulations in Switzerland, which made planning more difficult as lockdowns went into effect. To compare with another vital supply chain, entire food distribution channels like HORECA (HOtel/REstaurant/CAtering), and work cafeterias shut down, and consumers switched overnight to sourcing all of their food from supermarkets. The Coop and Migros grocery chains, however, responded admirably to this surge in demand, with only temporarily empty shelves (usually re-stocked overnight), and no life-threatening shortages. As the lockdowns were relaxed, the return to office work had the opposite effect on vaccine demand, which was expected to be correlated to the home address. This illustrates that what was missing in the Covid-19 vaccine supply chain was a strategic understanding of the target population, of who has been vaccinated, where, and why. The vaccination campaign unknowingly faced a population which was shifting daily (Figure 6), which, in turn, shifted demand. The cantonal vaccine distribution channels were designed, however, for an immobile population defined by residential maps.

The supply chain literature confirms that demand segmentation supports the design of a more responsive supply chain. In most countries, not only is the distance to a point of distribution a barrier to vaccination, but socioeconomic and psychological factors (norms, religion, education level), also influence the patient's willingness to be vaccinated (38, 42). Different groups of the Swiss population, which tend to live in identifiable parts of the canton, could have been targeted with more specific care options. Elderly patients tend to prefer treatments by familiar physicians, while younger patients are more open to public places, like shopping malls or mobile vaccination buses. In other words, the distribution channels (where to locate a vaccination center, and of which size), should have been designed to fit the characteristics of patients in the catchment area. The capacity needed per vaccination center could also have been adapted over time to fit the size of the population in the catchment area.

In a country where authorities diligently collect a volume of detailed statistical data on its population, the organization of a life-saving vaccine supply chain should be guided by analyses of demand for the purposes of both equity and reach. For this, Swiss authorities could draw upon information assets from their existing databases: density, age, socioeconomic status, income, and religion, as well as relevant behaviors like commuting trajectories (Figure 6). Using this information, simple and cost-effective measures, like individual invitations to be vaccinated, could have been issued, but they were not.

Finally we emphasize the need for collaboration within the cantonal public health authorities. For any emergency response to succeed, vaccine supply chain managers must address the same disease with the same vaccines for the same priority groups and have the same plan for a national pandemic response. Without insisting on centralized execution, there is no justification from a supply chain perspective for doing the same job 26 times over in each canton, rather than combining resources to work together. An exceptional global pandemic like Covid-19 poses an unheard-of threat to the national security of the Swiss population. A small country which has so many of its people crossing cantonal borders on a daily basis (Figure 6), should be coordinating decisions to fight an infectious disease on a bigger scale than the local level, to ensure an optimal – the fastest and most exhaustive – national response.

### Limitations

Like every study, this one has limitations, of which we will emphasize the most significant before proceeding to recommendations for the future. First and foremost, a supply chain which is designed to enable its strategic intent will exert no influence upon the patient's final decision to accept the vaccine. When assessing whether vaccination rates in Switzerland could be maximized, the scope of the study was restricted to the effects of the network design only. To use the terminology of Palagyi et al. (5) only the Hardware of EID (Surveillance, Workforce, Infrastructure, Medical Supplies, and Communication) and not its Software (Governance and Trust) were mapped. Once the system has been designed, there is work to be done to establish trust between the public and the particular healthcare initiative. The demand–supply matching function of a supply chain assumes that demand exists and in the case of vaccines, does not need to be generated (which does occur in commercial business cases). The results of this study therefore do not account for the effects of vaccine hesitancy, which were significant at the time. Second, the map which was drafted based upon the data is a snapshot of a system which necessarily continues to evolve over time, and may no longer reflect current realities (22). The authors acknowledge the improvements visible on the streets of Switzerland, like immunization buses. As in commercial supply chain management, mapping exercises should be repeated on a regular basis for diagnostic purposes and to support decision-making across organizational boundaries (24).

### Recommendations to Public Health Decision-Makers

In spite of the limitations, certain learnings will apply to vaccine health systems in Switzerland and elsewhere. Specifically, we recommend three interrelated initiatives: (1) a dashboard to centralize supply chain information from end-to-end, (2) cantonal collaboration on design and execution of the supply chain, and (3) evidence-based distribution planning.

First, the creation of a visual dashboard should combine pre-existing population data with newly collected Covid-19 specific data to support decision-making and assessing outcomes. Operations of the vaccination campaign should tap available geospatial data to derive insights into possible gaps and combine these with the pre-existing data about the population characteristics (like religious conviction), to analyse the root causes of vaccine hesitancy in different groups.

**Figure 7:**
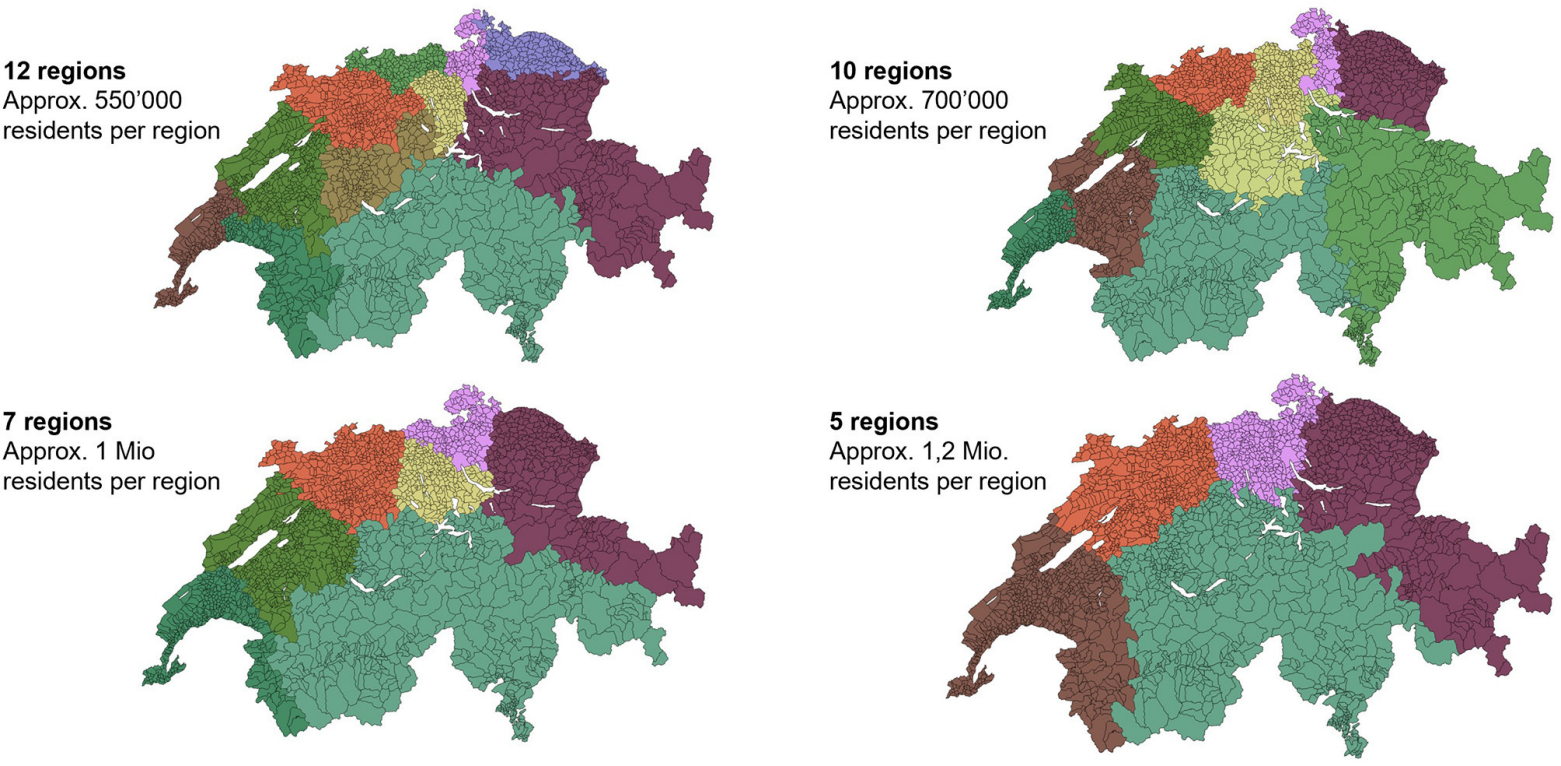
Four alternative definitions of equal catchment areas for vaccine distribution, based on residential densities. Maps like these could guide decisions on which municipalities/cantons should work together in order to maximize the reach of residential populations. Source: analysis and illustration by authors using data source (37).

Second, a differentiated picture of the target population can enable the segmentation of the supply chain into “service regions” (Figure 7) in which several cantons or municipalities work together. A reduction of the number of segments/service regions based on population distribution would be a more realistic model than the historical legacy of 26 administrative borders.

Third, the deeper understanding derived from the dashboard with geospatial data could be used to design distribution channels to maximize vaccination reach and rates. An overview of the distribution of priority groups within the catchment region allows administrators to plan how many doses are potentially needed when, as well as where. The most appropriate channel can be deployed to address the population in this region e.g., vaccination centers should the patients tolerate “fast and impersonal care,” or actively recruit primary care doctors should “more trust be needed.” Understanding the factors which drive demand helps to precisely allocate the scarce resources (which include skilled staff as well as dosages and injection material), required to swiftly vaccinate as many citizens as possible in an emergency.

The resources in Switzerland exist to achieve the strategic intent of the vaccine supply chain. The design and information flows must, however, be corrected to better support repeated booster vaccinations as the disease becomes endemic. Officials should learn now, and apply their experience in supply chain design, information sharing, and collaborative evidence-based analysis later to the routine management of public health (44). May this study deliver useful insights to support the necessary development.

## Author Contributions

BT-W set up and supervised the research project, worked with the stakeholders, and drafted the final manuscript based on inputs from the team. PB constructed the upstream section of the map and drafted inputs. PF constructed the downstream section of the map and drafted inputs. SW sponsored the project and reviewed and corrected the final results and manuscript. All authors contributed to the article and approved the submitted version.

## Funding

Open access funding was provided by ETH Zurich.

## Conflict of Interest

The authors declare that the research was conducted in the absence of any commercial or financial relationships that could be construed as a potential conflict of interest.

## Publisher's Note

All claims expressed in this article are solely those of the authors and do not necessarily represent those of their affiliated organizations, or those of the publisher, the editors and the reviewers. Any product that may be evaluated in this article, or claim that may be made by its manufacturer, is not guaranteed or endorsed by the publisher.
