# Erratum: Mapping the swiss vaccine supply chain

**DOI:** 10.3389/fpubh.2023.1178929

**Published:** 2023-03-14

**Authors:** Frontiers Production Office

**Affiliations:** Frontiers Media SA, Lausanne, Switzerland

**Keywords:** COVID-19, vaccine supply chain, supply chain mapping, GIS, Switzerland

An omission to the funding section of the original article was made in error. The following sentence has been added: “Open access funding was provided by ETH Zurich.”

The original article has been updated.

